# Heart murmurs in apparently healthy cats caused by iatrogenic dynamic right ventricular outflow tract obstruction

**DOI:** 10.1111/jvim.15774

**Published:** 2020-04-28

**Authors:** Luca Ferasin, Heidi Ferasin, Eoin Kilkenny

**Affiliations:** ^1^ Specialist Veterinary Cardiology Consultancy Alton, Hampshire United Kingdom; ^2^ Lumbry Park Veterinary Specialists – Cardiology Alton, Hampshire United Kingdom

**Keywords:** cardiology, cardiovascular, dynamic obstruction, echocardiography, feline, heart murmur, iatrogenic

## Abstract

**Background:**

Heart murmurs are detected commonly in apparently healthy cats during routine physical examination, and Doppler echocardiography ultimately is required to identify the source of flow turbulence causing the murmur. However, in some cases, the origin of the murmur cannot be identified on echocardiographic examination, even by experienced clinicians. The application of gentle pressure with the ultrasound transducer against the chest wall of a cat can induce temporary narrowing of the mid‐right ventricular (RV) lumen, causing blood flow turbulence even in the absence of cardiac abnormalities.

**Objectives/Hypotheses:**

To evaluate the effect of pressure of the ultrasound transducer against the chest wall of cats during echocardiography (provocative testing) on RV blood flow. The main hypothesis is that provocative testing can increase RV outflow velocity and cause flow turbulence. The second hypothesis is that the effect of this maneuver is independent of changes in heart rate during testing.

**Animals:**

Sixty‐one client‐owned, apparently healthy cats with heart murmurs on physical examination.

**Methods:**

Retrospective review of echocardiographic examinations of 723 cats presented for investigation of a heart murmur.

**Results:**

Outflow systolic velocity increased from 1.05 ± 0.26 to 1.94 ± 0.51 m/s during provocative testing (*P* < .0001); no correlation was found between RV outflow peak velocity and heart rate during provocative testing (*P* = .34; *r* = 0.1237).

**Conclusions and Clinical Relevance:**

Right ventricular outflow tract obstruction and associated heart murmur can be iatrogenically induced in apparently healthy cats by increasing pressure on the right chest wall with an ultrasound probe.

**Abbreviations**
Ao: aorta
DRVOTO: dynamic right ventricular outflow tract obstruction
LA: left atrium
LVH: left ventricular hypertrophy
RV: right ventricle/ventricular

## 1 INTRODUCTION

Heart murmurs are detected commonly in apparently healthy cats during physical examination, and the prevalence of heart disease in these patients varies from 16% to 77% depending on geographic location, examiners, and study methods.^1^, ^2^ The most common underlying cardiac abnormality observed in cats with incidentally detected murmurs is hypertrophic cardiomyopathy, reported in 15% to 62% of cases.^3^, ^4^, ^5^, ^6^, ^7^ However, non‐pathological causes of murmurs also have been described, with dynamic right ventricular (RV) outflow tract obstruction (DRVOTO) representing the most common cause (8%‐16%).^3^, ^4^, ^5^

Pathological and non‐pathological causes of heart murmurs however can coexist in apparently healthy cats, and these murmurs cannot be accurately assessed by auscultation alone.^1^, ^2^ Therefore, Doppler echocardiographic evaluation ultimately is required to identify the cause of blood flow turbulence responsible for this clinical finding.

Nevertheless, even Doppler echocardiography performed by experienced clinicians occasionally can fail to identify the origin of murmurs in cats, and failure to identify the cause of a murmur and classify it as either pathological or benign can lead to uncertain prognosis and unnecessary owner anxiety.^1^, ^2^

Over the last decade, we have incidentally observed that applying gentle pressure with the ultrasound transducer to the right side of the chest wall of a cat can induce temporary narrowing of the mid‐RV lumen, iatrogenically inducing DRVOTO and subsequently evoking blood flow turbulence. We also have observed that a similar phenomenon can be reproduced by gently pressing the stethoscope head against the right chest wall of the cat, inducing an audible murmur during auscultation. Therefore, our main objective was to evaluate the effect of increased pressure of the ultrasound transducer against the chest wall of apparently healthy cats during echocardiographic examination on RV outflow velocity. We hypothesized that such a maneuver would increase RV outflow velocity, ultimately resulting in blood flow turbulence in this anatomical area. We also hypothesized that this effect is independent of changes in heart rate during this test.

## 2 MATERIALS AND METHODS

Clinical records of echocardiographic examinations performed in cats for diagnostic investigation of a heart murmur at 2 institutions (Specialist Veterinary Cardiology Consultancy, SVCC Ltd and Lumbry Park Veterinary Specialists, LPVS) were reviewed retrospectively. Only apparently healthy cats without any signs of underlying cardiovascular or systemic disease were included in the analysis. Furthermore, only patients that had blood flow turbulence in the right infundibular tract induced by gentle pressure to the chest wall with the ultrasound transducer during image acquisition of the right parasternal short axis view at the level of the heart base (provocative testing) and that did not have any functional or structural abnormalities during echocardiographic examination were selected. Subjects that required sedation before echocardiographic examination were excluded from the study.

All echocardiograms were performed by a board‐certified cardiologist (L. F.) using an Esaote MyLab 30Gold Cardiovascular (MyLab, Esaote S.p.A., Genoa, Italy) (SVCC Ltd) or Esaote MyLabClass C (MyLab, Esaote S.p.A.) (LPVS) echocardiographic machine and a 7.0‐10.0 MHz phased array transducer. All echocardiographic examinations were reviewed using dedicated offline ultrasound imaging software (MyLab Desk, Esaote S.p.A.) to confirm the final diagnosis and exclude cardiac abnormalities or cases in which provocative testing was not performed. Complete routine transthoracic echocardiographic examination (2‐dimensional, M‐mode, color, and spectral Doppler studies) was performed in each cat in both right and left lateral recumbency.^8^ The presence of DRVOTO was confirmed as systolic color Doppler aliasing in the RV just cranial to the tricuspid valve and extending into the RV outflow tract, associated with spectral dispersion and a scimitar‐like appearance of the RV outflow profile on spectral Doppler study, as previously reported.^9^ Provocative testing was performed by applying gentle pressure to the right side of the chest wall of the cats with the ultrasound transducer while observing a temporary narrowing of the RV lumen and simultaneous change of blood flow from laminar to turbulent flow on color Doppler mode in the right parasternal short axis view at the level of the heart base. The RV outflow peak velocity was measured by pulsed‐wave spectral Doppler immediately before and during the provocative maneuver. Instantaneous heart rate during Doppler acquisition was measured using digital calipers based on the R‐R interval on a simultaneous ECG recording. Collected data were transferred to an electronic spreadsheet (Microsoft Office Excel 365, Microsoft Corporation, Redmond, Washington) and verified by 2 of the investigators (L. F. and E. K.) for accuracy.

### 2.1 Statistical analysis

All analyses were conducted using Medcalc (MedCalc Software, Ostend, Belgium) and a probability value of .05 was utilized as an indication of statistical significance. Data distribution was assessed for normality using the D'Agostino‐Pearson test. Normally distributed data were expressed as mean ± SD and nonparametric data were expressed as median (25th‐75th percentile). A within‐subject paired *t* test was used to test the hypothesis that the differences between the paired measurements of the right outflow peak velocities (before and during provocative testing) were different from zero. A within‐subject paired *t* test also was used to test the null hypothesis that the heart rate before and during provocative testing was zero. A Pearson correlation coefficient (*r*) was used to analyze the degree of association between RV outflow peak velocity and heart rate obtained during Doppler acquisition.

## 3 RESULTS

The database search identified 723 cats (SVCC, n = 426; LPVS, n = 297) that underwent echocardiographic examination for diagnostic investigation of a heart murmur between 2010 and 2018. One hundred fourteen (15.8%) of these cats had an echocardiographic diagnosis of DRVOTO but only 61 (8.44%) cats (37 males and 24 females) met all inclusion criteria for the study, having DRVOTO as the sole cause of their heart murmur and no cardiovascular abnormalities observed on full echocardiographic examination. Twelve different breeds of cats were represented with domestic shorthair cats being most frequent (n = 38), followed by domestic longhair cats (n = 5), Maine coons (n = 4), Bengals (n = 3), British shorthair cats (n = 3), Sphynx (n = 2), Norwegian Forest (n = 1), British blue (n = 1), Persian (n = 1), Siamese (n = 1), Siberian (n = 1), and Exotic shorthair (n = 1).

The median age of the cats was 8.0 (6.0‐9.3) years and the mean body weight was 4.5 ± 1.22 kg. The average murmur grade reported in the medical records was 2.4 ± 0.7 of 6.

None of the cats had echocardiographic evidence of left ventricular hypertrophy (LVH; all cats had myocardial wall thickness in diastole <6.0 mm on B‐mode), left atrial dilatation (all cats had left atrial‐to‐aorta ratio <1.6) or RV hypertrophy (subjective assessment).

No arrhythmias or conduction abnormalities were observed during simultaneous ECG monitoring. Records of blood pressure measurement only were available in 21/61 (34.4%) cats, with a mean value of 142.3 ± 21.7 mm Hg.

**FIGURE 1:**
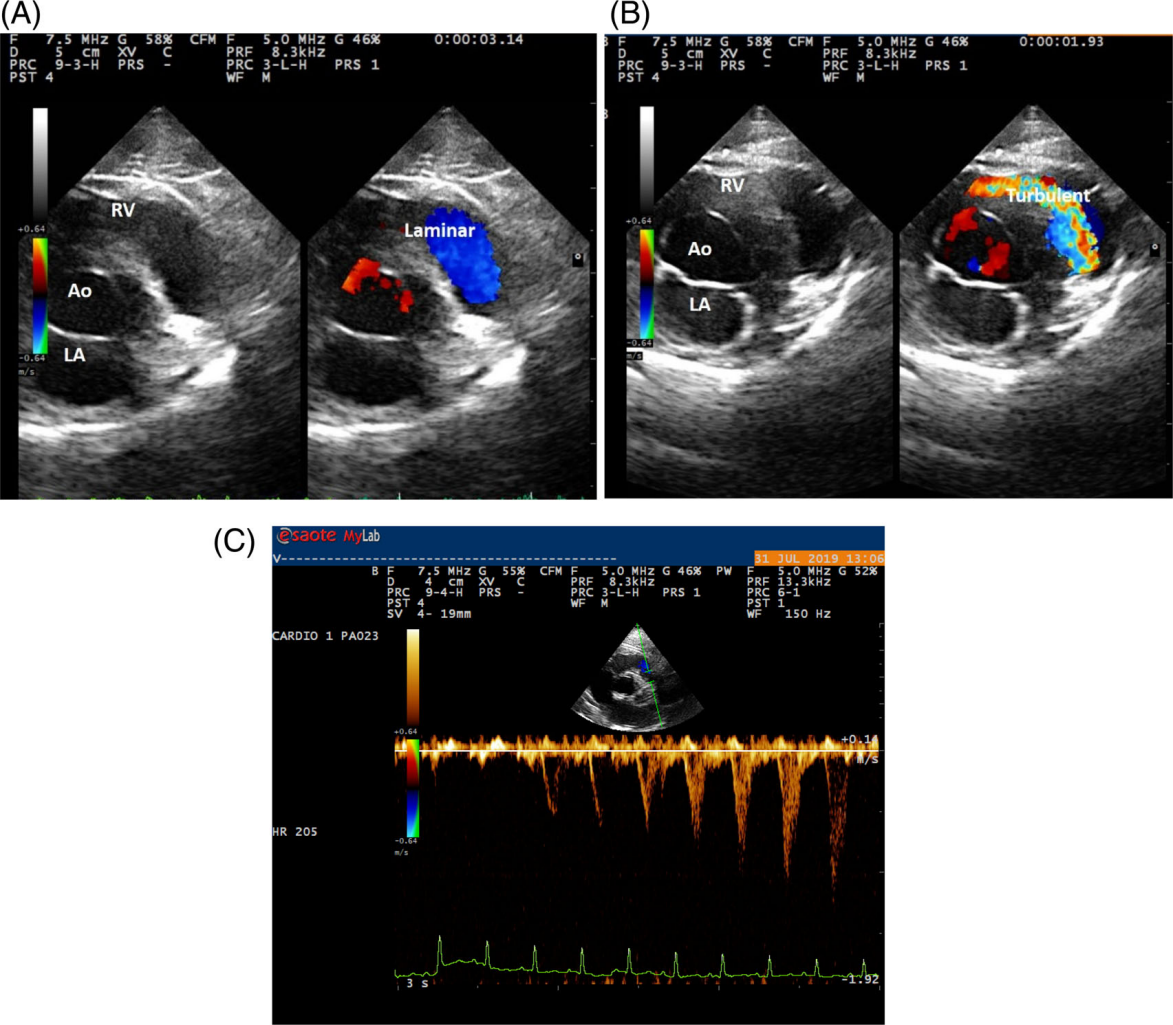
Echocardiographic images of a dynamic right ventricular outflow tract obstruction (DRVOTO) caused by provocative testing. All images were obtained from the right parasternal short axis view at the level of the heart base. A, Normal laminar flow (Laminar) was observed during systole in RV outflow tract prior to the maneuver. B, A turbulent flow (Turbulent) is observed in the same area as a result of provocative testing due to narrowing of the RV lumen caused by gentle chest compression with the ultrasound transducer. C, Spectral Doppler acquisition of the RV outflow showing the appearance of spectral dispersion, concave upstroke (scimitar‐like) with a late systolic increase of the peak velocity during the provocative maneuver (from 0.5 to 1.2 m/s). Ao, aorta; RV, right ventricle; LA, left atrium

All cats in the study had laminar blood flow on color Doppler assessment of the right infundibular tract, but turbulence could be visualized subsequently as variance on the color Doppler study after provocative testing. Similarly, provocative testing caused increased peak systolic velocity and a late‐peaking scimitar‐like appearance on spectral Doppler interrogation of the RV outflow tract, characteristic of dynamic mid‐systolic obstruction (Figure 1).

**FIGURE 2:**
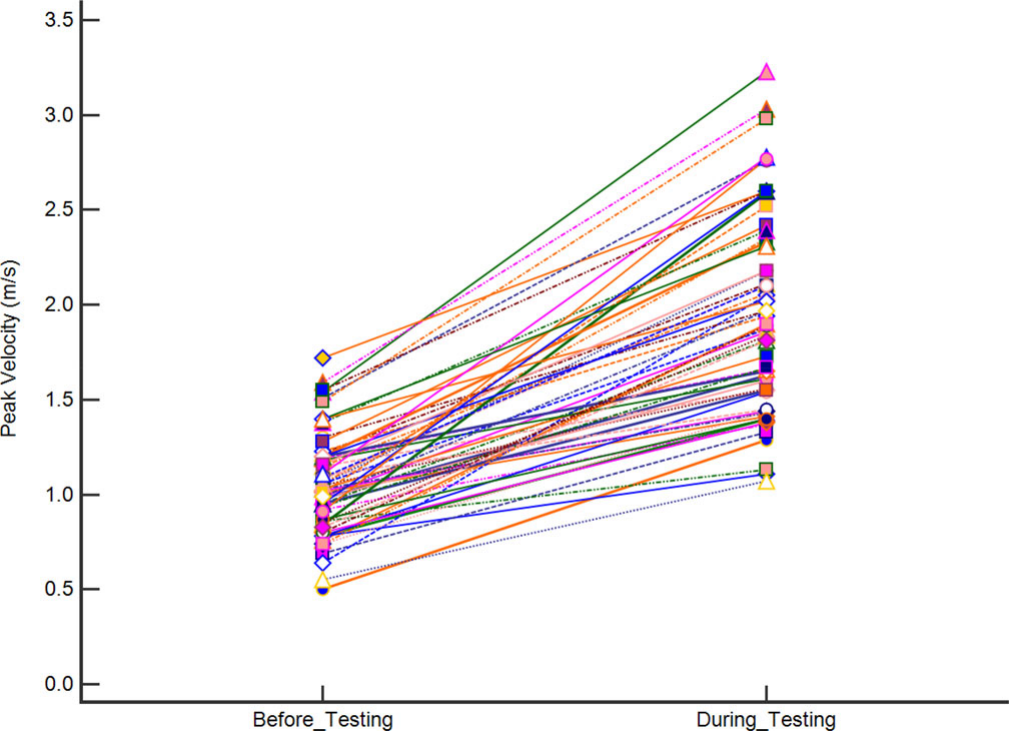
Effect of provocative testing on the right ventricular outflow peak systolic velocity reported as mean ± SD. Mean pre‐testing velocity was 1.05 ± 0.26 m/s and mean velocity during provocative testing was 1.94 ± 0.51 m/s. Their difference was 0.89 ± 0.40 m/s (*P* < .0001)

Outflow peak systolic velocities were normally distributed both pre‐testing (1.05 ± 0.26 m/s) and during provocative testing (1.94 ± 0.51 m/s) and their difference (0.89 ± 0.40 m/s) was statistically significant (*P* < .0001, Figure 2).

**FIGURE 3:**
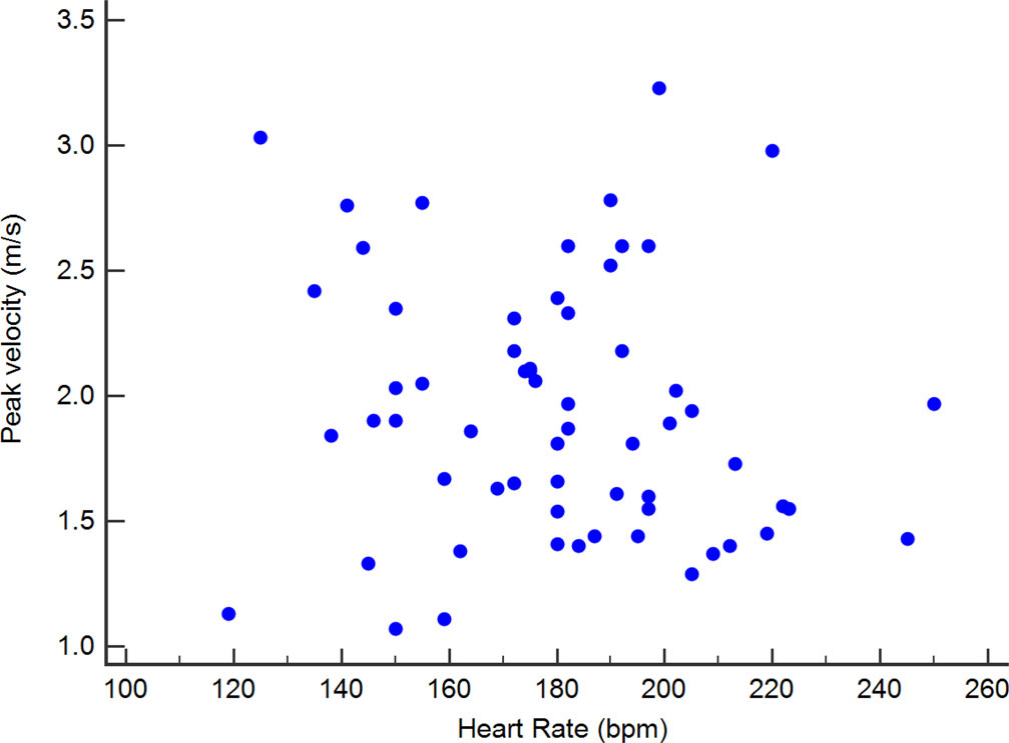
Scattered diagram showing the lack of correlation between heart rate and RV outflow peak velocity during provocative testing (*P* = .34; *r* = 0.1237)

Heart rate during provocative testing was 180.2 ± 27.6 bpm and no difference was found between heart rate measured before and during testing (*P* = .89). Furthermore, no significant correlation was found between RV outflow peak velocity and heart rate during provocative testing (*P* = .34; *r* = 0.1237; Figure 3).

## 4 DISCUSSION

We demonstrated that DRVOTO can be induced in apparently healthy cats by gentle compression of the right thoracic wall with the ultrasound transducer during echocardiographic examination (“provocative testing”). Because DRVOTO is a well‐described and universally accepted cause of heart murmurs in cats, it is likely that provoked DRVOTO was the cause of the murmur in these cats. Indeed, these apparently healthy cats did not have any structural or functional abnormalities on echocardiographic examination and therefore the origin of their heart murmur likely can be attributed to blood flow turbulence produced by the provocative testing (iatrogenic heart murmur).

Such compression tends to push the RV myocardial wall toward the interventricular septum, narrowing the anatomical region and eventually producing systolic outflow obstruction. In turn, this iatrogenic obstruction generates increased outflow velocity in the RV outflow tract, as indicated by aliasing on color Doppler evaluation and, on spectral Doppler study, spectral dispersion, and a scimitar‐like appearance of the RV outflow, similar to that observed during spontaneously occurring DRVOTO.^9^ Finally, increased blood flow velocity causes an increase in Reynolds number ($\mathrm{Nr}=\frac{\mathrm{\rho dv}}{\eta }$; *ρ* = blood density, *d* = blood vessel diameter, *v* = velocity, *η* = viscosity of blood) with a shift from laminar to turbulent flow, accompanied by an audible vibration of the affected area (murmur).^10^ Therefore, it is likely that such murmurs also can be caused by unintentional overzealous compression of the chest wall when the stethoscope head is used during cardiac auscultation. This type of inducible heart murmur in cats reported by one of the authors^11^ in 2012 but, to our knowledge, the occurrence of iatrogenic murmurs in cats has never been described previously in the veterinary literature.

Dynamic RV outflow obstruction is a relatively common cause of heart murmurs in cats, representing the sole cause of the murmur in 16% of cases in a study involving 57 apparently healthy cats.^4^ This result is almost identical to the percentage of cats with DRVOTO observed in our population (15.8%). In a different study, DRVOTO was reported in 8/16 (50%) of cats with heart murmur and 3 (18.7%) of these cats did not have echocardiographic evidence of heart disease, reflecting again the prevalence reported in our study.^5^

In the first report of DRVOTO in cats, heart murmurs were dynamic, varying in intensity with changes in excitement level and heart rate, and the velocity of the turbulent flow in the RV outflow tract consistently increased with increasing heart rate, and decreased when the heart rate decreased, often returning to normal laminar flow.^9^ However, this phenomenon was not observed in our study, as indicated by the non‐significant difference between heart rate measured pre‐testing and during testing and the lack of correlation between RV outflow peak velocity and heart rate during provocative testing. These observations suggest that, unlike spontaneously occurring DRVOTO, an adrenergic response was not associated with the dynamic obstruction observed in these cats.

**FIGURE 4:**
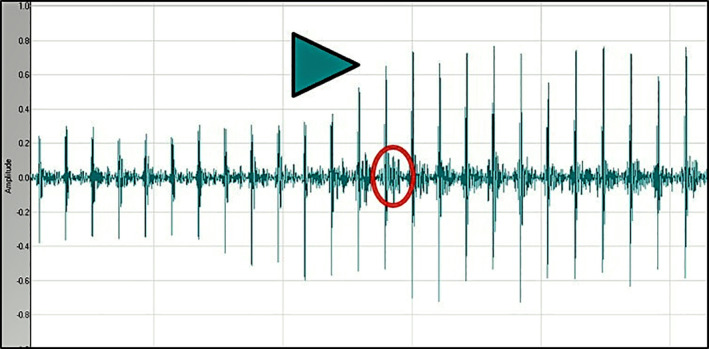
Phonocardiographic recording of a heart murmur induced in a cat in this study by gently pressing the stethoscope head on the right side of the chest wall. The maneuver starts at the level of the arrowhead, evoking increased amplitude of the heart sounds and appearance of a systolic murmur (circle)

Heart murmurs in apparently healthy cats are common and this phenomenon has been clearly reported in 2 large prospective studies involving cats from rehoming centers.^6^, ^7^ One of these studies described the presence of heart murmurs in 67/199 (34%) cats and 61 cats with murmurs underwent echocardiographic examination, which identified LVH in 18 to 62% of cats, depending on the echocardiographic criteria used for the classification of LVH.^7^ In the second study, involving 780 apparently healthy cats from 2 cat shelters, heart murmurs were auscultated in 318 cats (40.8%) and most of these murmurs (70.4%) were considered to be nonpathological.^6^ The presence or absence of DRVOTO in the above cat populations, however, was not recorded routinely, and some of the unexplained heart murmurs could have been caused by iatrogenic DRVOTO. A similar phenomenon also could have been evoked by pressing the stethoscope head against the chest wall, as illustrated by the phonocardiographic recording example in Figure 4.

The major limitation of our study is its retrospective design, and some relevant clinical details were not available for all 723 cats. For example, DRVOTO has been observed previously at a higher prevalence in cats with chronic kidney disease, systemic hypertension, anemia and hyperthyroidism but, although none of our patients had clinical evidence of systemic disease (apparently healthy), these clinical conditions could not be completely ruled out without additional diagnostic testing.

A second limitation is the fact that color Doppler aliasing and increased flow velocity cannot conclusively prove the origin of a murmur on echocardiographic examination. Although aliasing may not be an irrefutable explanation for the genesis of the murmur, the diagnosis of heart murmurs based on Doppler study is universally accepted, especially in patients in which structural lesions are not present or, at least, not easily identifiable.

A third limitation is the unknown force required to induce iatrogenic DRVOTO in cats. Ideally, a dynamometer would have been attached to the ultrasound transducer to accurately determine the force necessary to produce RV obstruction. However, very gentle force was sufficient to produce turbulence in the RV outflow tract without causing any discomfort or identifiable reaction in the cats, which also is reinforced by the stable heart rate observed throughout the provocative test. Therefore, this provocative testing can be considered both non‐intrusive and easily reproducible. Another limitation is the inability to determine if iatrogenic murmurs can be reproduced in all cats or whether this maneuver can only produce RV outflow obstruction in cats with particular body characteristics, such as a particular chest conformation or increased compliance of the chest wall. This possibility should be tested using a prospective study specifically designed to examine the effect on auscultatory findings of increased pressure of the stethoscope head in a larger and more heterogeneous population of cats.

Finally, heart murmurs in our study were not fully characterized. For example, although mid‐systolic murmurs would have been expected in these cats, the precise timing of the murmurs was not available in the clinical notes. This information would have been valuable to assist clinicians in distinguishing “iatrogenic murmur” from murmurs of different etiologies on cardiac auscultation.

In conclusion, we showed that nearly 1 in 10 apparently healthy cats presented for investigation of heart murmur can have iatrogenic RV obstruction without any concurrent cardiovascular abnormality. Such murmurs therefore can be considered benign in origin, being simply evoked by the pressure of the stethoscope head against the chest wall, and this information can provide valuable insight during diagnostic investigations of heart murmurs in these cats. Similarly, it is suggested that, if a heart murmur appears in response to increased pressure of the stethoscope head against the right side of the chest wall, such a finding should alert the clinician to the possibility that that murmur is likely to be iatrogenic.

## CONFLICT OF INTEREST DECLARATION

Authors declare no conflict of interest.

## OFF‐LABEL ANTIMICROBIAL DECLARATION

Authors declare no off‐label use of antimicrobials.

## INSTITUTIONAL ANIMAL CARE AND USE COMMITTEE (IACUC) OR OTHER APPROVAL DECLARATION

Authors declare no IACUC or other approval was needed.

## HUMAN ETHICS APPROVAL DECLARATION

Authors declare human ethics approval was not needed for this study.
